# Depression and anxiety during the perinatal period

**DOI:** 10.1186/s12888-015-0526-6

**Published:** 2015-08-25

**Authors:** Nichole Fairbrother, Allan H. Young, Patricia Janssen, Martin M. Antony, Emma Tucker

**Affiliations:** 1 Department of Psychiatry/Island Medical Program, University of British Columbia, Room 141, Eric Martin Pavilion, Royal Jubilee Hospital, 2328 Trent Street, Victoria, BC V8R 4Z3 Canada; 2 Centre for Affective Disorders, BRC Cluster Lead, Experimental Medicine & Clinical Trials Cluster, Department of Psychological Medicine, Institute of Psychiatry, Psychology and Neurosciences (IoPPN), King’s College London, PO72, De Crespigny Park, Denmark Hill, London, SE5 8AF Canada; 3 Maternal Child Health, UBC School of Population and Public Health, 2206 East Mall, Vancouver, BC V6T-1Z3 Canada; 4 Department of Psychology, Ryerson University, 350 Victoria Street, Toronto, ON M5B 2K3 Canada; 5 Faculty of Medicine, Island Medical Program, University of British Columbia, PO Box 1700 STN CSC, Victoria, BC V8W 2Y2 Canada; 6 Medical Sciences Building, University of Victoria, PO Box 1700 STN CSC, Victoria, BC V8W 2Y2 Canada

**Keywords:** Epidemiology, Perinatal, Mood disorders, AD, Reproduction

## Abstract

**Background:**

Mood and anxiety and related disorders (AD) account for a significant proportion of mental health conditions, with close to 30 % of the population (28.8 %) suffering from an AD at some time in their life, and over fifteen percent (16.2 %) suffering from a mood disorder. The existing empirical literature leaves a number of important gaps with respect to our understanding of mood, anxiety and stress related difficulties among pregnant and postpartum women. The objective of this research is to address these.

**Methods:**

Participants were 660 English-speaking pregnant women. Participants for the portion of the research estimating the prevalence/incidence of perinatal mood disorders and AD (*N* = 347) were recruited proportionally from a geographically defined area. All participants were recruited via prenatal clinic visits at hospitals, physician offices and midwifery clinics, and via community outreach at events and through word of mouth. Recruitment took place between November 9, 2007 and November 12, 2010. Participants were administered questionnaires prenatally at two time points (approximately 24 and 33 weeks gestation) and again at 4–6 weeks’ postpartum and 6-months postpartum. Prevalence/incidence study participants who screened above cut-off on one or more of the 4–6 week mood and anxiety questionnaires were also administered a diagnostic interview for mood disorders and AD at approximately 8–12 weeks postpartum.

**Discussion:**

This research addresses a number of gaps in our understanding of mood, anxiety and stress among pregnant and postpartum women. Specifically, gaps in our knowledge regarding the prevalence and incidence of (a) AD and mood disorders, and (b) anxiety and stress among women experiencing a medically high-risk pregnancy, interest in stress management training in pregnancy, mental health treatment barriers and access and screening for anxiety among pregnant and postpartum women are addressed. The findings from this series of studies have the potential to improve screening, assessment and treatment of mood and anxiety problems suffered by pregnant and postpartum women.

## Background

Mood and anxiety and their related disorders (AD) account for a significant proportion of mental health conditions, with close to thirty percent of the population (28.8 %) suffering from an AD at some time in their life, and over fifteen percent of the population (16.2 %) suffering from a mood disorder [1]. Half of all depressed patients also report symptoms meeting criteria for one or more AD [2]. Depression is the leading cause of disease-related disability among women [1], and women are also 1.6–1.7 times more likely to suffer from depression and/or AD during their lifetime than are men [3]. The perinatal period is of particular importance as maternal mood and anxiety difficulties are associated with adverse pregnancy outcomes, compromised parenting, impaired affect and behaviour regulation, and insecure attachment in offspring [3–9].

Anxiety during pregnancy is associated with adverse pregnancy outcomes such as miscarriage, preeclampsia, preterm delivery, and low birth weight [3, 10]. Further, children of highly anxious mothers have twice the risk for ADHD [11, 12]. Prenatal anxiety has been identified as a strong predictor of postpartum depression, even after controlling for prenatal depression levels [13–15].

In published reports of the prevalence and/or incidence of AD in pregnancy and/or the postpartum in which samples are either representative or unselected, and assessments are based on gold standard assessment methods (*i.e.*, diagnostic interviewing), the prenatal prevalence of AD ranges from 13 to 21 %, with the postpartum prevalence ranging from 11 to 17 % [16–19]. Postpartum incidence ranged from 2.2 to 8.8 %. None of these studies have included the full spectrum of the AD [18, 19]. A key objective of this research is to estimate the prevalence/incidence of the full spectrum of postpartum AD. If maternal AD are as common as they appear to be, then these disorders have serious negative consequences for a significant proportion of infants/children.

Depression and anxiety in pregnancy represent two of the strongest risk factors for postpartum depression [13, 20–22]. Similarly, postpartum depression is associated with significant emotional and marital distress as well as compromised physical and social functioning [23, 24]. Although a number of large-scale, high quality studies have assessed the prevalence of pre and postnatal depression (*i.e.*, an episode of major depression), using gold standard assessment methods (*i.e.*, diagnostic interviewing), to our knowledge, these estimates are based exclusively on episodic criteria or symptom severity; they are not based on the full diagnostic criteria for major depressive disorder [25, 26]. Consequently, it is likely that bipolar conditions contribute to the prevalence of postpartum depression. This possibility, to our knowledge, remains uninvestigated, and represents one of the objectives of the current research.

Screening for depression among postpartum women is routine in many places [27, 28]. The *Edinburgh Postnatal Depression Scale* (EPDS) is the most commonly used self-report instrument for the assessment of postpartum depression [29, 30]. Although the EPDS does contain items specific to anxiety, it is unknown whether the EPDS may be sufficient to detect the majority of women suffering from an AD [28, 31]. As a part of this study we have gathered data to determine if the current EPDS screening for depression is sufficient to detect AD also, or if there is a need for additional screening for anxiety.

Obstetrical complications, by definition, imply a threat to the health and well-being of the mother, her developing infant, or both [32, 33]. Over 20 % of all pregnancies involve obstetrical risks and account for up to 8000 births per year in British Columbia (BC), Canada alone [34]. While pregnancy can be a source of stress and anxiety for women who are experiencing normal, low-risk pregnancy, it is likely much more stressful and anxiety producing for women experiencing a pregnancy fraught with difficulties [35]. It is therefore likely that the prevalence of stress and AD among women experiencing a medically high-risk pregnancy may be even higher. Understanding the extent of stress and anxiety among high-risk obstetrical patients would provide extremely important information regarding the mental health needs of this group of vulnerable women. Despite the serious nature of medically high-risk pregnancies, which contribute to excess maternal and perinatal morbidity and mortality and corner a disproportionate among of health services expenditures, to date there have been no systematic studies of the prevalence of perinatal stress and anxiety among these women. This research aims to address this gap.

### Objectives

This research was multifaceted and involved several interconnected projects encompassing four broad areas of research: (a) perinatal AD, (b) perinatal mood disorders, (c) perinatal mental health treatment access, and (d) medically high risk pregnancy. The specific objectives within each of these areas are outlined below:

**Perinatal AD.**

1. Primary objectives: To determine:
  - the prevalence of maternal AD at 6–8 weeks postpartum, and
  - if additional screening beyond the EPDS is required in order to ensure adequate detection of AD among postpartum women.
2. Secondary objectives: To determine:
  i. the level of comorbidity of mood and AD at 6–8 weeks postpartum,
  ii. the temporal sequencing of AD and mood disorders during pregnancy and at 6–8 weeks postpartum, and
  iii. the course of anxiety symptoms from pregnancy to 6–8 weeks postpartum.

**Perinatal mood disorders.**

1. Primary objectives: To assess:
  - the contribution of the following to the prediction of depression (*i.e.*, symptom severity and diagnostic status) at 8-weeks postpartum:
    i. maternal, prenatal symptoms of the 6 primary AD (*i.e.*, social anxiety, specific phobias, obsessive-compulsive disorder, posttraumatic stress disorder, panic disorder and agoraphobia); and
    ii. maternal, prenatal symptoms of pregnancy specific stress.
  - the distribution of mood disorders (*i.e.*, major depressive disorder, minor depressive disorder and bipolar disorders) among postpartum women diagnosed with an episode of major depression.
2. Secondary objectives: To determine:
  - the prevalence of maternal postpartum depression at 6–8 weeks postpartum;
  - if the current, Province wide, self-report based screening for postpartum depression is sufficient to adequately detect AD among postpartum women, or if supplementary anxiety specific screening is needed; and
  - the course of mood symptoms from pregnancy to 6-months postpartum.

**Perinatal mental health treatment access.**

Assess 6-month postpartum mental healthcare access, use and cost among women diagnosed with mood disorders at 6–8 weeks postpartum.

**Medically high risk pregnancy.**

1. Primary objective: To assess the prevalence, nature and severity of anxiety and stress among women experiencing a medically high-risk pregnancy.
2. Secondary objectives:
  - To compare pre and postnatal levels of anxiety and stress among women experiencing a medically high-risk pregnancy with those of women experiencing a normal low-risk pregnancy;
  - To estimate the prevalence of persistent postpartum anxiety and stress among women who experienced a medically high-risk pregnancy; and
  - To assess the acceptability of and preference for prenatal stress management training among women experiencing a medically high-risk pregnancy.

## Methods

### Study design

Prospective cohort.

### Ethics

Ethical approval for the research was granted by the University of British Columbia/Children’s and Women’s Health Centre of British Columbia Research Ethics Board (UBC C&W REB). Consent for the completion of questionnaire packages was obtained from participants via online and/or mailed forms. A second consent was completed by all interview participants, for their participation in the interview portion of the research.

### Inclusion/Exclusion criteria

Study participants were classified as either: (a) those who met the inclusion/exclusion criteria for the full sample, and (b) those who met the more restrictive inclusion/exclusion criteria for the mood and AD prevalence sample. The inclusion/exclusion criteria for the prevalence sample were more restrictive as it was necessary to ensure representativeness of this portion of our sample of birthing women.

#### Full sample

All pregnant women fluent in English were eligible to participate.

#### Prevalence sample

Prospective participants were eligible to participate if they lived in the City of Vancouver, Canada at the time of recruitment, were pregnant, and spoke English fluently. The City of Vancouver was selected as the geographical boundary for the research.

### Recruitment

Pregnant women were recruited via prenatal clinic visits, physician offices and midwifery clinics at BC Women’s Hospital, St Paul’s Hospital and Burnaby Hospital, through community outreach at events and through word of mouth. Formal arrangements for recruitment were made with the appropriate individuals at each of the above-mentioned locations. Recruitment at these sites was primarily carried out via direct-approach (*i.e.*, approaching women as they waited for their appointments). The remainder were recruited passively through the use of posters and pamphlets. Recruitment took place between November 9, 2007 and November 12, 2010. A total of 668 women consented to participate.

### Representativeness (Prevalence Sample)

Based on 2003/2004 statistics, approximately 6000 babies are born to Vancouver residents each year (British Columbia Reproductive Care Program, 2004). Of these, 98 % of births by Vancouver residents take place at BC Women’s Hospital (73 %), Saint Paul’s Hospital (19 %), Burnaby Hospital (4 %), or at home (2 %). To maximize the representativeness of our sample, we recruited proportionally from each of the recruitment sites. Additionally, data weighting will be used during analysis, where appropriate, to ensure that our collected data are representative of the sites of birth of the general population of birthing women in Vancouver.

### Participants

#### Prevalence sample

There are 347 women in the prevalence portion of the research. Of these, 152 were eligible for interview based on their questionnaire responses. Of the 152 potential interview participants, 37 either declined to be interviewed (*n* = 7), were unresponsive to our attempts to schedule an interview (*n* = 9), or were not invited due to administrative error (*n* = 21). The final sample comprised 310 participants.

Of these, 76.3 % gave birth at BC Women’s Hospital, 16.3 % at Saint Paul’s Hospital, and 1.8 % at home. The remainder (5.7 %) gave birth elsewhere. On average, women were 27.3 weeks pregnant (*SD* = 8.5) at the time of enrollment. The vast majority were married (79.4 %) or living with a partner (16.6 %). The remainder were single (3.1 %), divorced (0.3 %), or separated (0.6 %). The majority of participants were Caucasian (72.4 %) or Asian (18.5 %). The remainder were First Nations Canadians, Hispanic, or Middle Eastern (3.4 %), or did not provide data regarding race/ethnicity (5.7 %). For 64.7 % of the sample, they were expecting their first child. Most were singleton pregnancies (94.3 %), and several were twin pregnancies (4.2 %).

#### Full sample

There are a total of 660 participants in the full study sample. Participants gave birth at BC Women’s Hospital (70.5 %), Saint Paul’s Hospital (9.3 %), home (3.0 %), Burnaby General Hospital (1.5 %), and other (11.7 %). The vast majority were married (80.3 %) or living with a partner (16.6 %). The remainder were single (5.1 %), divorced (0.3 %), or separated (0.3 %). The majority of participants were White/European (70.3 %) or Asian (22.6 %). The remainder were First Nations Canadians, Black, Hispanic, Middle Eastern, or Caribbean (5.3 %), or of mixed racial background. Over half (57.3 %) of the sample was expecting their first child. There were 594 singleton pregnancies, 34 twin pregnancies, and one set of triplets.

### Sample size

Our final prevalence sample size of 310 women will permit us to determine the prevalence of AD (assuming the prevalence to be approximately 15 %), with a 95 % confidence interval, to within ±3.97 %. Assuming the prevalence of perinatal depression to be approximately 6 %, the 95 % confidence interval would be accurate to within ±2.64 %.

### Screening

Women invited to participate in the study, were asked to complete up to four sets of screening questionnaires for anxiety and depression across pregnancy and the first 6-months postpartum. Most women entered the study between 30 and 32 weeks gestation, completing the first screening package at this time. Women who were recruited at or before 24 weeks gestation were administered an additional early prenatal questionnaire package. At 40-weeks’ gestation, participants were mailed the same screening package and asked to complete and return it between 4 and 6 weeks postpartum. Women who were residents of Vancouver at the time of recruitment and scored above predetermined cut-offs on any of the mood and anxiety measures, completed at 4–6 weeks postpartum, were telephoned and invited to participate in the interview portion of the study.

### Interviews

Project interviewers conducted diagnostic assessments of all participants who (a) scored above cut-off on any of the postpartum screening measures, (b) were resident of the City of Vancouver at the time of study enrolment and (c) consented to participate in the interview portion of the study. In total 123 of the 310 women in the prevalence sample were interviewed. Diagnostic assessments took place between 6 and 8 weeks postpartum. Women were offered the choice of coming to BC Women’s Hospital for their interview, or having the interview conducted in their (the participant’s) home. The vast majority (*i.e.*, > 90 %) elected to have the interview conducted in their home. At the time of the diagnostic assessment, women with symptoms meeting criteria for any mood or AD were provided with appropriate mental health referrals.

### Follow-up assessment

At 6-months postpartum, all participants were sent follow-up questionnaires to assess:

i. Current status of mood and anxiety symptoms.
ii. Mental health care received since the birth of their baby.
iii. Appropriateness of the care based on the 4-week postnatal diagnostic assessment.
iv. Out of pocket cost for mental health care.
v. Accessibility of mental healthcare received.

### Assessment tools

#### Demographic and reproductive measures

Included in the initial questionnaire package were demographic questions including age, income, education, and marital status. Information about past and current medical and reproductive history was also obtained. In later questionnaire packages participants were asked for information about changes in health status as well as labour and delivery experiences. These questions included a mixture of multiple choice and open-ended questions as needed.

#### Self-report measures

The following measures were used to assess for symptoms of all six AD and for depression. Women completed each of these screening tools at each study time point and returned them by mail to the investigators. These measures have been selected because they are psychometrically sound and possess good sensitivity and specificity.

##### Generalized anxiety disorder 7-item scale (GAD-7) [36]

the GAD-7 s a 7-item self-report measure designed to assess for generalized anxiety disorder (GAD) [36]. Items are rated on a 0–3, Likert-type scale. Higher scores are indicative of higher levels of symptoms of GAD. The GAD-7 has been found to demonstrate good reliability as well as convergent (with worry, anxiety, low mood and stress), criterion, construct, factorial, and procedural validity [36, 37]. The GAD-7 is also sensitive to change over time [37]. Compared with the EPDS, the GAD-7 was more reliable and valid in identifying GAD among pregnant. The optimal cut point for optimizing sensitivity and specificity has been found to be 10 [36]. Consequently, we selected a cut score of 10 for this study, representing a sensitivity of 89 % and a specificity of 82 %.

##### Obsessive compulsive inventory – revised (OCI-R; Foa, *et al*., 2002) [38]

the OCI-R is an 18-item self-report measure of symptoms of OCD. Items are scored on a 5-point, Likert-type scale from 0 to 4. The factor structure of the OCI-R indicates a 6-factor model with 3-items in each factor [38]. These factors represent the following content domains: Washing, Checking, Obsessing, Hoarding, Ordering, and Neutralizing. The psychometric properties of the OCI-R are excellent [38–41]. When used as a screening tool to detect OCD, the OCI-R obsessing scale shows higher levels of sensitivity (74 %) and specificity (76 %) compared with the full OCI-R (66 % for sensitivity and 63 % for specificity) [38]. Consequently, we have used the OCI-R obsessing subscale as our measure of OCD. The OCI-R obsessing subscale shows good internal consistency with coefficient alphas ranging from 0.77 to 0.89 [38, 39]. We used a cut-off score of 4 on the obsessing subscale. This represents a sensitivity of 74.4 % and a specificity of 76.1 %.

##### Mini social phobia inventory (Mini-SPIN) [42]

the Mini-SPIN is a brief, 3-item measure derived from the full scale *Social Phobia Inventory* (SPIN) [43]. Mini-SPIN items were selected from the full scale SPIN as follows. The authors selected the SPIN items which best discriminated between those with generalized social anxiety disorder and controls. To do this, the three items with the greatest difference in mean score between those with generalized social anxiety and controls were selected. Items are scored on a 0 (not at all) to 5 (extremely) Likert-type scale. A cut-score of 6 on the Mini-SPIN has been found to represents good sensitivity (88.7 and 93.8 %) and specificity (89.9 and 63.6 %) [42, 44]. The Mini-SPIN has been found to demonstrate strong internal consistency reliability, as well as convergent and discriminant validity [44].

##### Panic disorder self-report (PDSR) [45]

the PDSR is a hierarchical questionnaire designed to screen for panic disorder, modeled following the panic disorder module of the Anxiety Disorder Interview Schedule (ADIS-IV) [46]. The PDSR begins with questions key to a diagnosis of panic disorder. Only if these initial questions are answered in the affirmative, are the remaining questions administered. The initial four items assess the recurrent and unexpected nature of panic attacks, followed by three items assessing worry that attacks will recur, and changes in behaviour in response to the panic attacks. The final 12 items assess symptoms of the attacks and their interference with life. A score of 8.75 has been found to optimize sensitivity and specificity and was therefore selected for this research [45]. At 8.75, the PDSR demonstrates a sensitivity of 89 % and a specificity of 100 %. The PDSR has been found to have excellent test-retest reliability, and convergent and discriminant validity [45].

##### Mobility inventory for agoraphobia (MI) [47]

the MI is a 27-item self-report inventory of avoidance behaviours and panic attacks in agoraphobia. The measure assesses agoraphobic avoidance on two scales, one when the person is alone and the other when accompanied. Items are scored on a 1 (rarely avoids) to 5 (always avoids) Likert-type scale. Test-retest reliability has been shown to be .75-.86 for the accompanied subscale, and .75-.90 for the alone subscale [48]. A number of cutoff scores have been suggested for the MI, however we chose an average of alone and accompanied score of 1.5 as the cutoff. This score has been shown to have a sensitivity of 78 % and specificity between 76 and 85 %. When using the avoidance alone scale with a cutoff score of 1.5 the sensitivity is 91 % and specificity is 67 % [49].

##### Specific phobia questionnaire (SPQ) [50, 51]

the SPQ (Fairbrother & Antony, 2011) is a 43-item, self-report measure of specific phobias. Each item assesses a specific fear (*e.g.*, dogs, elevators, driving in new places) and includes a rating for fear and for interference. The fear and interference ratings are scored on a 0 (none) to 4 (extreme), Likert-type scale. The measure has been administered in the current study as well as to a clinical sample of over 700 individuals diagnosed with one or more AD, and a student sample of approximately 200. To date, the SPQ fear of dogs item has been found to correlate well with the *Dog Phobia Questionnaire* (*r* = 0.73 for fear and 0.60 for interference) [50]. Further psychometric analysis of the SPQ is currently underway. In the current study, we used a cut-off score of 6 or greater (combined fear and interference ratings) on one or more SPQ items.

##### PTSD checklist (PCL) [52]

the PCL is 17-item self-report measure to assess symptoms of posttraumatic stress disorder (PTSD). Items are rated on a 1 (not at all) to 5 (extremely) Likert-type scale. Total possible scores range from 17 to 85. The PCL has been found to have good internal consistency, test-retest reliability, and convergent validity [53]. For this study, we selected a cutoff score of 44, as this maximizes sensitivity (94 %) and specificity (86 %). This is compared to a cutoff score of 50 which has a sensitivity and specificity of 78 and 86 %, respectively [52].

##### Edinburgh postnatal depression scale (EPDS) [29]

The EPDS is a 10-item self-report measure screening tool for postnatal depression. The sensitivity and specificity of the EPDS are in acceptable ranges (65–100 %, and 49–100 %, respectively) [54]. Higher sensitivity relative to specificity is appropriate for a screening instrument. The EPDS is the most widely used screening tool for postpartum depression [55].

#### Diagnostic instrument

The *Structured Clinical Interview for DSM-IV* (SCID-IV) [56] is a well-validated structured diagnostic interview designed for the assessment of a wide range of psychiatric problems including all the mood and AD. The SCID was used to assess:

i. AD.
ii. Mood disorders.
iii. Adjustment disorders.
iv. History of psychiatric problems.
v. History of mental health service utilization (*e.g.*, psychiatric hospitalizations, pharmacological and psychosocial interventions for psychological problems and diagnoses previously assigned).

### Analysis

Data will be analysed using SPSS Version 22.

#### Perinatal AD

We will provide 95 % confidence intervals for all prevalence/incidence estimates of maternal perinatal anxiety and mood disorders. *Z*-tests will be used to compare the sensitivity of depression screening alone in detecting cases of AD, compared to the sensitivity of a combination of depression and anxiety screening tools. Information about the level of mood and AD comorbidity, and the timing of disorder onset will be presented descriptively. We will use one-way repeated measures analysis of variance to examine changes in severity of symptoms of AD from pregnancy to 6 months postpartum.

#### Perinatal mood disorders

Multiple linear regression will be used to assess the unique contribution of symptoms of the six primary AD and pregnancy-specific stress to the prediction of postpartum depression. Established risk factors for postpartum depression will be entered as covariates in the first block. Mood disorder prevalence/incidence estimates will be presented via 95 % confidence intervals based on diagnostic interview data. We will use one-way repeated measures analysis of variance to examine changes in severity of symptoms of depression from pregnancy through to 6-months postpartum.

#### Perinatal mental health treatment access

Information about participants’ postpartum use of mental health services, difficulties in access, the match between their diagnosis and the health care they received, and the out-of-pocket cost of these services will be presented descriptively.

#### Medically high risk pregnancy

Ninety-five percent (95 %) confidence intervals will be presented for all group means and prevalence estimates. Between-group comparisons based on continuous questionnaire data will be analyzed using one way ANOVA. Between group comparisons of the prevalence of AD stratified by maternal risk will be analyzed using a chi-squared test of independence. Interest in stress management training provided during pregnancy will be presented descriptively, and compared across risks groups using one way ANOVA.

### Current status

Data collection for the study is complete. The data set has been cleaned, and a substantial portion of the data analysis has been completed. Manuscript preparation is currently underway.

## Discussion

Substantial empirical, clinical and policy-directed attention has been given to perinatal depression. In contrast, significantly less attention has been given to perinatal AD [15]. This is surprising, particularly in light of the fact that AD are likely more common among pregnant and postpartum women than is depression [16]. Despite the attention given to perianal depression, little of this work has investigated the underlying diagnosis of sufferers (*i.e.*, whether depression occurs in the context of a major depressive disorder, a bipolar disorder, or other mood disorder). The overarching goal of this series of interconnected studies is to increase our understanding of the scope and nature of perinatal mood and anxiety and disorders.

This research includes the first comprehensive (*i.e.*, encompassing all of the AD) study of maternal perinatal AD, using gold standard assessment procedures, carried out to date. This will also be the first report of a systematic study of the prevalence and course of perinatal stress and anxiety among women experiencing a medically high-risk pregnancy. Although significant attention has been given to understanding of the causes and consequences of postpartum depression, a number of significant knowledge gaps remain. One area of particular importance is that of the relationship between perinatal depression and anxiety. Although we now know that prenatal anxiety and the presence of a prenatal AD significantly increase the risk of postpartum depression, the specific contribution of individual domains of anxiety has yet to be investigated. This represents one of the key objectives of the current research. A further neglected area is that of the role of bipolar conditions in postpartum depression. To our knowledge, investigations of postpartum disorder prevalence have yet to assess the frequency with which postpartum depression occurs in the context of an underlying bipolar disorder. Our immediate goal is to improve our knowledge in these two key areas. Finally, we are also assessing the utility of the EPDS as a stand-alone screening tool for perinatal AD.

In summary:

1. We will estimate the prevalence and incidence of AD among pregnant and postpartum women. This information will (a) make it possible to decide where to direct treatment efforts for this population and (b) provide necessary data for a large-scale epidemiological study of perinatal AD incidence/prevalence.
2. We will estimate the prevalence of anxiety and stress among women experiencing a medically high-risk pregnancy. This research will provide critical information regarding the mental health needs of high-risk obstetrical patients, information which will guide the development and implementation of prevention and treatment programs for this group of vulnerable women.
  We will also assess the level of interest of these women in receiving stress management training in pregnancy and the preferred delivery format for this intervention (*i.e.*, in person, via audio or visual materials, or workbook). Should the level of interest merit it, we will proceed to the development of a stress management treatment module for delivery to this population of women.
3. Among women who are diagnosed with a mental health condition in pregnancy and or the postpartum we assess whether they access mental health services for their condition(s), difficulties in access, out of pocket costs and the degree to which the treatment accessed represents an evidence-based approach to their difficulties. Cognitive behaviour therapy frequently represents the treatment of choice for many of the AD and depression. Sadly, this approach to treatment is often difficult to access, or is very costly [57]. We are interested in learning of the challenges women face when seeking treatment for perinatal mood and anxiety difficulties (*e.g.*, difficulty locating services, costs associated with the services), and the frequency with which women receive treatment which is recognized as an evidence-based approach to their difficulties. What we learn from this research has the potential to impact healthcare funding decisions.
4. Data collected in this study will provide important information regarding the distribution of mood disorders (*e.g.*, major depressive disorder, bipolar disorders) among women who have experienced an episode of perinatal depression, as well as provide information about risk factors for postpartum depression; this information which will contribute to the development and implementation of prevention and treatment programs for perinatal women.
5. Our findings will also indicate the number of cases of maternal AD detected using the EPDS compared to the number detected using additional self-report measures of anxiety. This information will allow us to determine if additional screening, beyond the EPDS, is needed in order to adequately detect maternal postpartum AD.

### Limitations

While this study aims to fill the gaps left by other research in the field we acknowledge that this research also suffers from some of the same limitation as previous work. Specifically, although we were successful in recruiting proportionally within the defined geographic area for the prevalence portion of the research, and our initial recruitment was above our target, the study experienced a higher loss to follow-up than anticipated and concluded with a smaller than ideal sample size for some aspects of the project (*e.g.*, prevalence estimates for individual AD). Further, participants were largely self-selected and therefore our sample is not likely to be fully representative of the population from which it is drawn. With respect to the prevalence sample, proportional recruitment and data weighting were used to mitigate differences between the population and the sample.

Because diagnostic interviews were conducted in the postpartum only, the data for the prevalence/incidence of mood and AD in pregnancy is based on participant retrospective self-report and may therefore be less reliable than the postpartum estimates. For the prevalence/incidence portion of the research, only those women who screened above cut-off on self-report measures of depressed mood and anxiety were invited to be interviewed makes it likely that some cases of anxiety and/or depression were missed. Consequently, prevalence/incidence estimates for mood and anxiety and related conditions are likely an underestimate of actual population rates.

It is also important to note that this study was conducted prior to the publication of the DSM-5 and therefore uses definitions and criteria from the DSM-IV which may lead to differences in comparing with future studies.

## Conclusions

The findings from this series of studies has the potential to add significantly to our understanding of maternal perinatal mood and AD, stress and anxiety among women experiencing a medically high risk pregnancy, as well as treatment preferences and barriers to access among pregnant and postpartum women. The aim is to translate our findings into improved screening, assessment and treatment of mood and anxiety problems suffered by pregnant and postpartum women.

## Abbreviations

AD: Anxiety and related disorders (*i.e.*, anxiety disorders, and obsessive-compulsive and related disorders, and trauma and stressor related disorders)
ADHD: Attention Deficit Hyperactivity Disorder
ADIS-IV: Anxiety Disorders Interview Schedule for the DSM-IV
BC: British Columbia
C&W: Children’s and Women’s Health Centre of British Columbia
DSM-IV: Diagnostic and Statistical Manual for DSM-IV
DSM-5: Diagnostic and Statistical Manual for DSM-5
EPDS: Edinburgh Postnatal Depression Scale
GAD-7: Generalized AD
MI: Mobility Inventory for Agoraphobia
OCD: Obsessive-Compulsive Disorder
OCI-R: Obsessive-Compulsive Inventory – Revised
PCL: PTSD Checklist
PDSR: Panic Disorder Self Report
PTSD: Posttraumatic Stress Disorder
REB: Research Ethics Board
SCID-IV: Structured Clinical Interview for DSM-IV
SPIN: Social Phobia Inventory
SPQ: Specific Phobia Questionnaire
SSRI: Selective Serotonin Reuptake Inhibitor
UBC: University of British Columbia
